# Correction: Stability of gum Arabic-gold nanoparticles in physiological simulated pHs and their selective effect on cell lines

**DOI:** 10.1039/c8ra90090f

**Published:** 2018-12-05

**Authors:** Heloise Ribeiro de Barros, Mateus Borba Cardoso, Carolina Camargo de Oliveira, Célia Regina Cavichiolo Franco, Daniel de Lima Bellan, Marcio Vidotti, Izabel C. Riegel-Vidotti

**Affiliations:** Grupo de Pesquisa em Macromoléculas e Interfaces, Departamento de Química, Universidade Federal do Paraná – UFPR CxP 19032 CEP 81531-980 Curitiba PR Brazil izabel.riegel@ufpr.br; Brazilian Synchrotron Light Laboratory P.O. Box 6154 13083-970 Campinas Brazil +55 19 3512-1004; Departamento de Biologia Celular, Universidade Federal do Paraná – UFPR CxP 19081 CEP 81531-980 Curitiba PR Brazil

## Abstract

Correction for ‘Stability of gum Arabic-gold nanoparticles in physiological simulated pHs and their selective effect on cell lines’ by Heloise Ribeiro de Barros *et al.*, *RSC Adv.*, 2016, **6**, 9411–9420.

The authors regret that the name of one of the authors (Daniel de Lima Bellan) was shown incorrectly in the original article. The corrected author list is as shown above.

The Royal Society of Chemistry apologises for these errors and any consequent inconvenience to authors and readers.

## Supplementary Material

